# Overcoming Barriers to Family Planning through Integration: Perspectives of HIV-Positive Men in Nyanza Province, Kenya

**DOI:** 10.1155/2013/861983

**Published:** 2013-04-30

**Authors:** Rachel L. Steinfeld, Sara J. Newmann, Maricianah Onono, Craig R. Cohen, Elizabeth A. Bukusi, Daniel Grossman

**Affiliations:** ^1^Department of Obstetrics and Gynecology and Reproductive Sciences, University of California, San Francisco (UCSF), 50 Beale Street, Suite 1200, San Francisco, CA 94105, USA; ^2^Center for Microbiology Research, Kenya Medical Research Institute (KEMRI), Mbagathi Road, P.O. Box 19464, Nairobi 00202, Kenya; ^3^Ibis Reproductive Health, 17 Dunster Street, Suite 201, Cambridge, MA 02138, USA

## Abstract

This study explored barriers to and facilitators of using family planning services among HIV-positive men in Nyanza Province, Kenya. From May to June 2010, in-depth interviews were conducted with 30 men receiving care at 15 HIV clinics. The key barriers to the use of family planning included concerns about side effects of contraceptives, lack of knowledge about contraceptive methods, myths and misconceptions including fear of infertility, structural barriers such as staffing shortages at HIV clinics, and a lack of male focus in family planning methods and service delivery. The integration of family planning into HIV clinics including family planning counseling and education was cited as an important strategy to improve family planning receptivity among men. Integrating family planning into HIV services is a promising strategy to facilitate male involvement in family planning. Integration needs to be rigorously evaluated in order to measure its impact on unmet need for contraception among HIV-positive women and their partners and assure that it is implemented in a manner that engages both men and women.

## 1. Introduction

Many studies have demonstrated the diversity and complexity of reproductive intentions among people living with HIV in sub-Saharan Africa, which are influenced by personal, health-related, sociocultural, socioeconomic, and gendered factors [1–6]. Often HIV infection changes but does not eliminate fertility desires [1]. Some HIV-positive individuals believe that having children gives them reasons to live [1, 7, 8], and some bear children to avoid raising suspicions of HIV infection [8, 9]. Still others want to replace children who have died due to HIV [1, 8, 10]. However, many HIV-positive individuals want to avoid pregnancy due to financial reasons and being satisfied with the number of children they have [6]. Other deterrents to having children include fears of orphaning a child, of vertical transmission of HIV, and of infecting a negative partner during conception [1, 6, 7]. Several studies have shown that fertility desires differ by gender, that one partner's fertility intentions can impact the other's, and that men and women are influenced differently by community opinions regarding HIV and reproduction [1, 5, 8, 11, 12].

Unmet need for contraception and unintended pregnancy are prevalent among HIV-positive women and couples in sub-Saharan Africa [13, 14]. Based on evidence of cost-savings and demonstrated effectiveness of contraception in averting HIV-positive births [15, 16], the World Health Organization/United Nations Population Fund Glion Call to Action emphasizes family planning as one of four critical elements of a comprehensive prevention of mother-to-child transmission (PMTCT) of HIV strategy [17]. Integrating family planning into HIV care and treatment is being promoted by international public health agencies, local organizations, and some governments, including the government of Kenya, to ensure that HIV-positive individuals have access to comprehensive contraceptive counseling and services [18].

Traditionally, family planning programs have been directed towards women, since it is women who become pregnant and the majority of family planning methods are used by women. Moreover, women are more frequently in contact with the health care system because of their overall responsibility for family health, especially for the health of infants and children under five years of age. However, men are key decision-makers around use of contraceptives [6], and studies have shown that men usually want to be involved in reproductive decision-making [6, 19]. It has been shown that when men are involved in family planning, there are improvements in uptake of contraception [20]. Furthermore, reproductive health programs that target couples have been shown to be more effective at increasing contraceptive use than those directed to individuals [19, 21]. The move toward integrating family planning services into HIV care and treatment may offer an opportunity to engage with men and their partners to increase contraceptive uptake. Despite the growing body of literature about the complex reproductive desires of people living with HIV [8], there exist few in-depth studies about patient perspectives of integration of family planning and HIV services and even fewer focused solely on HIV-positive men.

The Kenyan government, in accordance with international policy, has demonstrated a strong commitment to improve linkages between reproductive health and HIV/AIDS services, reflected in its recently developed national strategy for reproductive health and HIV/AIDS integration [18]. This qualitative study was conducted in Nyanza Province, the region in Kenya with the highest prevalence of HIV and high unmet need for family planning, as part of formative research for a cluster-randomized controlled trial (RCT) evaluating the impact of integrating family planning services into HIV care and treatment clinics on contraceptive prevalence (http://clinicaltrials.gov/, NCT01001507). We sought to explore the barriers and facilitators to the use of family planning services among HIV-positive men accessing HIV care and treatment in public sector clinics in rural Kenya. Our aim was to gain a better understanding of HIV-positive men's experience with and needs for family planning in order to guide efforts to integrate family planning into HIV care and reduce unmet need for contraception among people living with HIV.

## 2. Material and Methods

### 2.1. Sites

This qualitative study was conducted between May and June 2010 as part of baseline data collection for a cluster randomized controlled trial evaluating the impact of integrating family planning into HIV services on contraceptive prevalence. Participants were recruited from 15 public sector HIV treatment clinics taking part in the RCT in the Kisumu East, Nyatike, Rongo, and Suba Districts of Nyanza Province including two dispensaries, eight health centers, three subdistrict hospitals, and two district hospitals. All sites were supported by Family AIDS Care and Education Services (FACES), a collaboration between the University of California, San Francisco (UCSF) and the Kenyan Medical Research Institute (KEMRI) [22]. All sites provided comprehensive HIV care and treatment including the provision of antiretroviral therapy (ART). At the time of this study, none of the sites were offering specific couples-based counseling or ART services, although such services may have been provided on a case-by-case basis. The study was approved by the Committee on Human Research at UCSF and the Ethical Review Committee at KEMRI.

### 2.2. Eligibility

Eligible participants were nonsterilized, HIV-positive men 18 years and older accessing care at one of the participating HIV clinics. A convenience sample of two men per site was selected to participate. The interviewer screened male patients after they completed their clinical visit and invited the first eligible and willing participant to complete the interview. After the first interview was completed, this process was repeated to identify the second participant. Each participant provided voluntary written informed consent and received a reimbursement of approximately $2.50 USD.

### 2.3. Open-Ended Interviews

Thirty open-ended interviews were conducted to explore male clients' family planning preferences in the context of integration of family planning into HIV care and treatment services. Interviews were conducted in participants' first language (Dholuo) by a trained interviewer. Each interview lasted approximately 60 minutes and was based on a semistructured interview guide. The domains covered in the interview guide included reproductive intentions, perceived barriers to obtaining and using effective contraception, and acceptability of various family planning services, including integrated with HIV care and home-based services. All interviews were audio recorded.

### 2.4. Data Analysis

Interviews were transcribed and translated into English. Data were managed in Atlas-ti 6.0 (Scientific Software Development, Berlin), and transcripts were coded and analyzed using a grounded theory approach [23]. Two investigators independently conducted the initial coding of a sample of transcripts according to a codebook constructed from the interview guide content and a preliminary content analysis of the raw data; inductive codes based on the data were developed as concepts emerged. Discrepancies were resolved through discussion and consensus. An iterative process was used to develop the final qualitative analysis codebook which allowed for refinement of our analysis and thematic concepts. In the final analysis, codes and quotations were grouped to identify thematic trends and variant views. Quotes presented here are identified by the age of the participant, the number of living children, disclosure of HIV status to the primary partner, and the HIV status of the primary partner. These characteristics were chosen as they may impact use of contraception and decisions about accessing family planning services in an HIV clinic.

## 3. Results

The demographic characteristics of participants are shown in Table 1. For those whose age was known, about half were 35 or older, and half younger. All but one was married. Most had primary school education or less, and about half were literate. The median number of living children was four. On average, men had been diagnosed with HIV two years previously, and the majority was on antiretroviral therapy. Nearly half of men (43%) reported using condoms alone and another 27% reported using condoms with another method of family planning, and a minority (36%) said their partner was using a modern method of contraception (Injectable, combined oral contraceptives, or female sterilization). Among the 30 participants, 21 (70%) reported their wife or main partner was HIV+, three (10%) reported she was HIV−, four (13.3%) reported that they did not know the status of their wife or main partner, and the remaining were missing or not applicable. The majority of the individuals reported having disclosed their HIV status to their wife or main partner (*n* = 24, 80%). Three men had not yet disclosed their status and three had missing data or were not applicable.

### 3.1. Relevance of Family Planning

Many HIV-positive men, independent of their fertility desires, understood the importance of planning one's family to improve the health of the mother and child, to enable them to better care for their children, and for financial reasons. For instance, one man stated that “*…it [family planning] enables you to plan your family and only have a child when you want, that enables you to take good care of the child…*” (29 years, 3 children, disclosure missing, HIV+ partner). The importance of child-spacing was a common theme. One man described it in the following way: “*in the culture of the Luo community if a child is closely followed by another pregnancy then the child will grow weaker and sicker.*” (42 years, 7 children, disclosed to partner, HIV+ partner.) Another man discussed spacing children in the following manner: “*…we needed a break to first take care of the other children we have and push them ahead before getting another child. Having several of them would make it difficult to meet all their needs at once, for instance one needs clothing, another one school fees, hospital fees*…” (30 years, 5 children, disclosed to partner, HIV+ partner).

### 3.2. Barriers to Family Planning

Despite the fact that many participants understood the importance of planning one's family, the use of more effective contraception, such as hormonal, intrauterine, or permanent methods, was low. Several barriers to family planning use emerged as predominant themes in the interviews, including concerns about side effects of contraceptives, lack of knowledge about contraceptive methods, myths and misconceptions, structural barriers such as staffing shortages, and a lack of male focus in family planning methods and service provision. Some of the concerns and perceived barriers mentioned are based on actual experiences or their partner's experiences, while others are hypothetical concerns or based on common attitudes and beliefs in the community.

#### 3.2.1. Side Effects of Contraceptives

Side effects of contraceptives were a common concern, specifically irregular bleeding. One man said: “*I have heard that at times when a woman is on family planning medications they have longer periods and the flow of blood never ceases… As a man, at times you want to have sex, but you realize she has blood, yet she was on her period just the other day*.” (Age missing, 4 children, disclosed to partner, HIV+ partner.)

#### 3.2.2. Lack of Knowledge and Myths and Misconceptions

Family planning educational talks were routinely given in the waiting area of the HIV clinic where these men received HIV services. Most men were able to name at least two family planning methods. However, many men were not aware of long acting reversible contraceptives and permanent methods. There remains a particularly large gap in knowledge related to vasectomy, an infrequently utilized form of contraception in this community. Fear of infertility due to nonpermanent forms of contraception was a concern repeated by some men, “*I have also heard rumors that the drugs can make somebody never able to get pregnant again.*” (Age missing, 4 children, disclosed to partner, HIV+ partner.) However, some men were more knowledgeable about the variety of contraceptive options available, including the importance of dual protection. For instance, one man said: “*the condom helps to protect against many other things and not just pregnancy. Therefore, as much as my wife uses injections, we also use condoms*.” (29 years, 3 children, disclosure missing, HIV+ partner.) Throughout the interview, this man stated that he and his wife have achieved their desired family size. He also stated: “*my wife is also infected and whenever we have sex we do what we were told at the hospital to reduce the effect of HIV…we must use condoms to avoid injuring the other person by adding more HIV…*” Many other men with both HIV-positive and HIV-negative partners discussed the importance of condoms for HIV prevention and as illustrated here, “to avoid adding HIV” or acquiring a second strain of the virus.

#### 3.2.3. Structural Barriers

A few men were aware of challenges that their partners may be facing in accessing family planning services. One challenge that was repeated a few times was shortage of staff to provide services. One man stated: “*on the day she went, she didn't get the services as the provider was away. When she went the next time she was still away, that happened on four different times then on the fifth visit she got the services*.” (42 years, 7 children, disclosed to partner, HIV+ partner.) Another mentioned that when there are stockouts of commodities, his wife must go to the pharmacy to buy the medication and have someone administer the injection. Another man reported that condoms are sometimes out of stock at the health facility, and he must buy them.

The cost of the services did not appear to be a barrier for most men because family planning services are often provided for free or a small fee. One stated: “*I think it is just okay, because they must use a syringe; however the medicine is only 30 shillings [$0.35]*” (36 years, 2 children, disclosed to partner, HIV+ partner). When referring to the cost of condoms, one man said: “*there are condoms that are sold; they are the ones I have paid for, however most of the times I use the ones given for free at the health centers*.” (37 years, 4 children, disclosed to partner, HIV− partner.)

Although the majority of men reported that they lived close by and are able to walk to the health facility, distance to the clinic was reported by a few men as a barrier. “*The clinic is a bit far from us, such that if I come on foot, it takes about 2 1/2 hours to reach here*.” (37 years, 4 children, disclosed to partner, HIV− partner.) Another said: “*the terrain is what complicates it, you see this place has a lot of hills*.” (42 years, 7 children, disclosed to partner, HIV+ partner.) Wait time at the clinic was reported as a barrier for one individual. Only one of the men interviewed had accompanied his spouse to get family planning services, and therefore they, unlike the female clients, were not accustomed to going to two separate clinics to receive family planning and HIV/AIDS care services.

#### 3.2.4. Lack of Male Options for Family Planning

Other than male condoms, the most common contraceptive methods are used by women. Several HIV-positive men noted that they would like to have their own methods for birth control. Lack of male options for family planning services was reported as a barrier for a few men in this population. One man said: “*you see us as men, our options are limited, it is the women who have a variety of options to choose from*…” (37 years, 4 children, disclosed to partner, HIV− partner). Another man stated: “*there are times I hear presentations over the radio on family planning services and where the services are, however personally I have not gone to see for myself since it is mostly women who visit those clinics*...” (29 years, 3 children, disclosure missing, HIV+ partner). Several men mentioned they would like to accompany their partner to access family planning services so that they could learn more about the services.

### 3.3. Facilitators of Family Planning Related to Integration of Family Planning and HIV Services

#### 3.3.1. A Sense of Belonging and Community at the HIV Clinic

When men were asked about the possibility of receiving family planning services in the HIV clinic, there was a general agreement among most men that integration would improve access to family planning services among HIV-positive men and their partners. Convenience and improved continuity of care were mentioned as supportive reasons for integration of family planning services. One man stated: “*since we are already HIV-positive and getting our medication at the PSC [HIV clinic], if those services [family planning] can be offered at the PSC then I would prefer to get them from the PSC*.” (Age missing, 6 children, disclosed to partner, HIV+ partner.)

Men appeared to feel a sense of belonging at the HIV clinic. One said: “*personally I would prefer to go to the patient support center [HIV clinic] because it is our clinic; I wouldn't want to go to the maternity clinic. It is at the patient support center that I will have to tell them my problems because it is my place and they are our people.*” (30 years, 5 children, disclosed to partner, HIV+ partner.) Another said: “*I thought the clinic for children is only meant for those carrying small children.*” (Age missing, 11 children, disclosed to partner, HIV− partner.) “*I like it there [HIV clinic] because I am used to that place, the care providers at the clinic also talk to us freely and are concerned about our health, the other clinic only deals with women and children's issues, but here I will find fellow men and women, and we can talk freely.*” (42 years, 11 children, disclosed to partner, HIV+ partner.) Access to service providers and speed of services also seemed to be a facilitator for receiving family planning services at the HIV clinic. “*Because here [the HIV clinic] is a busy place, and when you come you are likely to find people. But in the other clinics…you may even find it closed and there are times when patients go back home without treatment because both the nurse and the doctor are away.*” (42 years, 7 children, disclosed to partner, HIV+ partner.)

A few men's opinions about integration of family planning and HIV services were indifferent, as one stated that: “*these are wings of the hospital; PSC [HIV clinic] is one of them so it is just the same as going to the maternal and child health clinic.*” (Age missing, 7 children, disclosed to partner, HIV+ partner.) While a few preferred family planning services to be delivered at the family planning clinic, the great majority of men were supportive of receiving family planning services in the HIV clinic.

#### 3.3.2. Faith in and Comfort with HIV Care Providers

Many men reported trust and confidence in the providers at the HIV clinic. One stated: “*I have confidence in the people working there [HIV clinic]. Again they have the qualifications and experience hence would give me good advice.*” (38 years, 1 child, disclosed to partner, HIV− partner.) Another said: “*because when one is HIV-positive, they need close attention, and if your wife gets pregnant and you are both positive, you get to know that the child needs to be given birth to at the hospital…*” (29 years, 3 children, disclosure missing, HIV+ partner). Another stated: “*because they are the ones who know and will understand my problems and hence will handle me well.*” (27 years, 1 child, disclosed to partner, HIV+ partner.)

Men often said that health care providers influenced their opinions about family planning. One man stated: “*it is mainly due to the counseling we receive here at the hospital and as a result of that I have realized that I am able to decide on the number of children to have and when to have them.*” (42 years, 11 children, disclosed to partner, HIV− partner.) Another man when discussing receiving family planning counseling said: “*you see when people openly discuss something, then stigma reduces and there is more acceptance as you realize you are not alone in your situation.*” (Age missing, 6 children, disclosed to partner, HIV+ partner.)

#### 3.3.3. Opportunity for Couples Counseling at the HIV Clinic

Family planning couple counseling provides an opportunity for a facilitated discussion to enable both partners to make an informed choice about contraceptive options that satisfies their personal, reproductive, and health needs. Integration of HIV and family planning services could facilitate couple counseling. A few men reported that health care providers could counsel couples who disagreed about their fertility preferences and family planning use. When asked about differences in fertility preferences, one man said that “*I had brought my wife to the clinic and the doctor talked to us together, we were counseled and she accepted the idea of family planning.*” (36 years, 3 children, disclosed to partner, partner HIV+.) Another man discussed his desire to accompany his partner to her family planning visit by saying “*I would like that because it gives us an opportunity to both talk to the doctor, she can give her opinion and I can also give my opinion as we get advised together.*” (30 years, 5 children, disclosed to partner, HIV+ partner.) Although this desire to accompany one's partner to a family planning counseling session was echoed by many men, only one man reported previously attending the FP clinic with his wife. Several men were informed that if they want to have a child they should speak with the health care provider before conceiving so that they can appropriately time the pregnancy and prevent mother-to-child transmission of HIV.

## 4. Discussion

Our study qualitatively explored views among HIV positive men in western Kenya about the idea of integrating family planning into HIV care and treatment. Despite commonly cited barriers to uptake and use of contraception, such as insufficient knowledge about contraceptive methods, fear of social disapproval, and fear of side-effects and health concerns [24–26], we found that most men in this exploratory study felt that integrating family planning into HIV care would be a preferable way to receive family planning information and services compared to separate services at the maternal and child health or family planning clinics. We found that many expressed a desire to learn more about family planning and felt that integrating family planning into HIV services would increase their knowledge about and involvement in family planning. Many also expressed feelings of confidence in the HIV care providers and a sense of belonging in the HIV clinic which may facilitate the likelihood that men and their partners would access family planning services, such as education and counseling, and personally adopt or encourage their partner to adopt new contraceptive methods if provided at the HIV clinic.

Men accessing HIV care and treatment reported theoretical ways in which integrating family planning into HIV care would make access to information and use of family planning easier for them and their partners. Many men in this study wanted to cease or delay having children, and as a result they were receptive to information that they received during the family planning educational talks about the various contraceptive methods. Although the majority of contraceptive methods are used by women, studies have shown that when men are involved in family planning discussions, there are improvements in uptake of contraception among women [20]. There is also a potential for HIV-positive men to gain knowledge about and access to vasectomy services. The impact of integration of family planning and HIV/AIDS services and the effect of health care provider counseling and education appeared to positively impact male acceptance and potential utilization of family planning by them and their partners.

Based on information gathered during the baseline interviews with male and female patients accessing HIV care, we designed the intervention to attempt to meet their family planning needs. The intervention included the provision of a range of family planning services within the HIV clinic. The services included family planning education, counseling, and contraceptive method provision including condoms, oral and injectable contraceptives, implants, intrauterine devices, and referral for tubal ligation and vasectomy services. Prior to the intervention, condoms were available in the HIV clinic, and clients interested in other methods were referred to the family planning clinic. Several months prior to the in-depth interviews, lay counselors had begun to deliver family planning educational talks in the waiting area of the HIV clinic.

Among this population of HIV-positive men, the most common barriers to supporting their partner's use of contraception included concerns over side effects, lack of knowledge of contraception methods, and myths and misconceptions including fear of infertility. Some, but not all, of the barriers to family planning uptake cited by men could be addressed through strengthened family planning education, counseling, and service provision within the HIV clinic.  A  related  baseline study conducted among HIV-positive women at the same HIV clinics in Kenya found that women reported similar barriers to family planning. The main difference was that women often stated that they did not use family planning or used it clandestinely due to partner opposition to family planning [27]. However, surveys of men and couples show that men are more likely to report contraceptive use than women, and men and women often have similar attitudes about family planning [28]. Misperceptions by women of husbands' attitudes, indicating absence of discussion, might be a more significant barrier to use.

Currently condoms, withdrawal, periodic abstinence, and vasectomy are the only options that require use or cooperation by men. These methods, with the exception of vasectomy, do not require interaction with the family planning service delivery system or a provider. A few men reported the need for more male-focused approaches and talked about the frustration that there were no more reversible male-controlled methods. The fact that we are still far from the development of a widely used, effective, and acceptable reversible male contraceptive [29] perpetuates male exclusion from family planning services and utilization of family planning. Additionally, the perception that the maternal and child health clinic is only for women and children has been raised as a barrier for male involvement in a variety of reproductive health services, including family planning and PMTCT [30]. Integration of family planning into HIV care may prove to be a way to allow more men to receive family planning education and counseling in a more neutral, less gender-biased way.

Family planning education provided in the waiting area of the HIV clinics reached both men and women. When HIV and family planning services are integrated, couples accessing HIV care in the same clinic could also attend family planning couple counseling sessions within the HIV clinic. We believe this has the potential to increase the number of men who participate in family planning discussions with their partners; however, since this is a baseline study, we do not know how it will impact use of contraception. Unfortunately, couple-based ART services are not routinely provided in this setting, unless upon request. We did not measure the extent that couples were accessing ART services together.

Inclusion of men in family planning counseling and education, however, is not enough to overcome the gendered power dynamics that impact contraception use. Differences in perceptions of gender equitability and gendered-power based differentials within relationships have been found to impact contraception use by women in western Kenya [31]. In order to successfully involve men in integrated family planning and HIV services, programs must be developed that take into account men's fears and vulnerabilities related to family planning. As has been seen in the field of HIV prevention [32, 33], it is not enough to focus on the empowerment of women to use condoms, or more effective contraception in this case, but programs must work towards transforming traditional gender roles and expectations in order to create a more egalitarian and supportive basis from which men and women can be empowered to make family planning decisions together.

Many of the study findings are relevant to all Kenyan men living in the region, independent of one's HIV status, such as concerns over side effects, lack of knowledge of contraceptive methods, and myths and misconceptions. Gendered-power-based differentials within relationships are also common in the general population. However, HIV infection may impact fertility desires, and concerns over HIV infection to children and partners may influence contraceptive choices. Of course, one important difference is that HIV-positive men accessing HIV care and treatment are already having regular contact with a clinical service, which may not be the case for HIV-negative men. We were particularly interested in whether these HIV-positive men in care would be receptive to receiving education about family planning, as well as services for them and their partners, in this setting.

Although providing family planning services in a location where men are already accessing HIV services will increase the likelihood of reaching men, a more couple-oriented approach to family planning is also needed. Many men reported their interest in attending their partner's family planning clinic visit, but it is yet to be seen if integrated services will improve couple-based counseling and decision making.

Additional strategies to improve the number of men who engage in family planning services include recruiting males as family planning providers and offering more family planning counseling for couples at home. HIV-positive men and women who are using contraceptive methods could be invited to contribute to family planning education talks at the clinic and in the community to promote female-oriented methods with men and vice versa. This is particularly important given that although clinic staff might be respected for their technical competence, the experiences of friends and family with contraceptive methods are far more influential [34, 35]. Additional efforts are also needed to provide balanced education and counseling, recognizing varying fertility desires. It is also important to recognize that some men and couples may need services to help them get pregnant using safer conception practices, noted by one man regarding his wife's repeated miscarriages and pregnancy complications. Although the majority of men in this study had disclosed their HIV status to their primary partner or spouse and majority of the men were in seroconcordant relationships, it is critical to tailor educational and counseling message to the individual couple's needs because disclosure and the HIV serostatus of the couple may impact their fertility desires and interest and use of contraception.

This exploratory study has several limitations. There may be a sample selection bias, and the men interviewed here may have differed in their attitudes towards accessing or utilizing family planning services within the HIV clinic than other men. Most men in our study had never accompanied their spouse to a family planning visit, and therefore most of the concerns and perceived barriers stated are hypothetical or based on actual experiences reported by their spouse. Furthermore, our participants were already accessing HIV care; we did not include HIV-positive men from the community not in care, or men of unknown HIV status, who may have different perspectives. Given that these men were recruited from clinic sites and might have associated interviewers with care providers, there is a risk that their responses were influenced by social desirability bias. Though theoretical saturation was reached around all main themes, some concepts could not be fully developed due to the small sample size. For example, since the majority of participants had not accessed formal family planning services, we could not fully assess perceived provider attitudes and/or family planning-related stigma. Finally, nuances of language and nonverbal communication strategies may have been lost or misinterpreted during the process of data transcription and translation.

Integration is a promising strategy that needs to be rigorously evaluated to measure the impact on unmet need for contraception among HIV-positive women and their partners. This study supports integrating family planning into HIV care and suggests that men would like family planning services available in the HIV clinic and that the HIV clinic provides a unique environment by which to facilitate male involvement in family planning. As countries roll out family planning integration into HIV clinics, it will be important to ensure that these programs develop in a manner that reaches both men and women with family planning information and services and provides a space for couples to discuss fertility intentions and contraception.

## Figures and Tables

**Table 1:** Participant characteristics (*N* = 30).

	*N* (%)
Age, mean (range)	33.5 (27–42)
18–34	12 (40%)
35–42	12 (40%)
Missing*	6 (20%)
Marital status	
Married	29 (97%)
Unmarried	1 (3%)
Education	
Primary school or less	19 (63%)
Secondary school or higher	7 (23%)
Missing*	4 (13%)
Literacy	
Reads with difficulty or not at all	13 (43%)
Reads easily	14 (47%)
Missing*	3 (10%)
Disclosure of HIV status to wife or main partner	
Disclosed	24 (80%)
Did not disclose	3 (10%)
Not applicable (no wife or partner)	1 (3%)
Missing*	2 (7%)
HIV status of wife or main partner	
HIV-positive	21 (70%)
HIV-negative	3 (10%)
Unknown to male participant	4 (13%)
Not applicable (no wife or partner)	1 (3%)
Missing*	1 (3%)
Number of living children, N = 29, median (range)*	4 (0–11)
Time since HIV diagnosis (years), *N* = 26, mean (range)*	2.0 (<1–7)
Currently on ART (*N* = 27)*	16 (59%)
Current contraceptive use—self or partner (*N* = 30)	
Injectable contraceptives	8 (27%)
Combined oral contraceptives	2 (6%)
Female sterilization	1 (3%)
Condoms	21 (70%)
Condoms only	13 (43%)
Condoms + other method	8 (27%)
No modern method	3 (10%)
Abstinence	3 (10%)

*Data were missing for some participants.
